# Mmu-miR-1894-3p Inhibits Cell Proliferation and Migration of Breast Cancer Cells by Targeting Trim46

**DOI:** 10.3390/ijms17040609

**Published:** 2016-04-22

**Authors:** Li Zhang, Xiaoying Li, Wei Dong, Caixian Sun, Deyu Guo, Lianfeng Zhang

**Affiliations:** 1 Key Laboratory of Human Disease Comparative Medicine, Ministry of Health, Institute of Laboratory Animal Sciences, Chinese Academy of Medical Sciences & Peking Union Medical College, Beijing 100021, China; zhangl@cnilas.org (L.Z.); leexyhb@126.com (X.L.); dw130032@126.com (W.D.); booboocx@163.com(C.S.); 2 Laboratory of Animal Sciences, Xuanwu Hospital of Capital Medical University, Beijing 100053, China

**Keywords:** breast cancer, mmu-miR-1894-3p, tripartite motif containing 46, metastasis, migration

## Abstract

Breast cancer is the second leading cause of cancer death in women and the presence of metastasis significantly decreases survival. MicroRNAs are involved in tumor progression and the metastatic spreading of breast cancer. Here, we reported that a microRNA, mmu-miR-1894, significantly decreased the lung metastasis of 4TO7 mouse breast cancer cells by 86.7% in mouse models. Mmu-miR-1894-3p was the functional mature form of miR-1894 and significantly decreased the lung metastasis of 4TO7 cells by 90.8% in mouse models. A dual-luciferase reporter assay indicated that mmu-miR-1894-3p directly targeted the tripartite motif containing 46 (Trim46) 3′-untranslated region (UTR) and downregulated the expression of Trim46 in 4TO7 cells. Consistent with the effect of mmu-miR-1894-3p, knockdown of Trim46 inhibited the experimental lung metastasis of 4TO7 cells. Moreover, knockdown of human Trim46 also prohibited the cell proliferation, migration and wound healing of MBA-MD-231 human breast cancer cells. These results suggested that the effect of knockdown of Trim46 alone was sufficient to recapitulate the effect of mmu-miR-1894 on the metastasis of the breast cancer cells in mouse and that Trim46 was involved in the proliferation and migration of mouse and human breast cancer cells.

## 1. Introduction

Breast cancer is the second leading cause of cancer death in women worldwide, with 232,670 new cases and 40,000 breast cancer deaths annually [1]. Cancer metastasis has a strong influence on the length of survival. The unraveling of metastasis mechanisms could give rise to novel therapeutic approaches to combat this disease [2].

MicroRNAs (miRNAs) are non-coding RNAs with gene expression regulatory functions, whose de-regulation has been documented in almost all types of human cancer, with respect to the non-tumoral tissue counterpart. miRNAs can function either as oncogenes or tumor suppressors in mammary tumor progression and tumor metastasis [3,4,5,6,7].

The latest version of miRbase (ver.21) reports included 1881 human miRNA sequences and 1193 mouse miRNA sequences (http://www.mirbase.org) [8]. Previous studies frequently used xenogeneic mouse models of transplanted human cancer cells into immunosuppressed mice to explore the role of human miRNA and their targets in breast cancer metastasis [9]. However, compared with xenogeneic mouse models, syngeneic models have more advantages, such as good reproducibility of the experimental tumor histology and growth rate and, most importantly, the full immunoreactivity of the host [9,10].

Here, we aimed to investigate the role of some conserved mouse miRNAs [11,12,13,14,15,16,17,18,19,20,21,22,23] with human homolog related to human cancer and several unconserved mouse miRNAs identified recently [24] in the metastasis of breast cancer cells in mice with full immunoreactivity, with the hope of finding new metastasis suppressor miRNA or new target genes. In this study, the effect of mmu-miR-1894 [24] and its target gene tripartite motif containing 46 (Trim46) on tumor metastasis was determined in mouse models.

## 2. Results

### 2.1. miR-1894 Is a Novel Metastasis Suppressor MicroRNA for Mouse Breast Cancer

**Figure 1:**
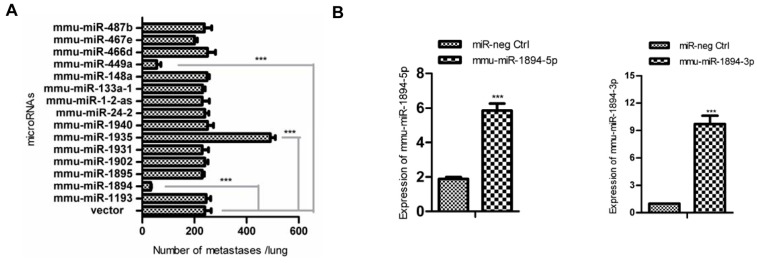

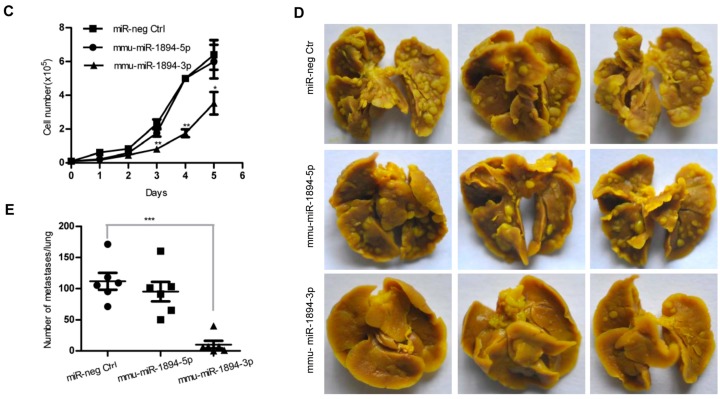
Mouse microRNA screening and lung metastasis assay. (**A**) The numbers of metastasis nodules in lungs were calculated 14 days after transplantation with the indicated microRNA or empty vector in 4TO7 cell lines (*n* = 6 for each group). *** indicates *p* < 0.001 *versus* vector control; (**B**) The expression of mmu-miR-1894-3p and mmu-miR-1894-5p in 4TO7 stable cell lines was determined by qRT-PCR. U6 snRNA was used for normalization. *** indicates *p* < 0.001 *versus* control; (**C**) Growth curves of the 4TO7 stable cell lines expression of mmu-miR-1894-3p and mmu-miR-1894-5p at indicated time. * indicates *p* < 0.05, ** indicates *p* < 0.01 *versus* control; (**D**) Representative photos for lung metastasis nodules. 4TO7 cells expressing mmu-miR-1894-3p or mmu-miR-1894-5p were injected into the tail veins of Balb/c female mice. Two weeks later, the lungs were fixed in Bouin’s solution and photographed; and (**E**) The numbers of metastasis nodules in (**D**) were calculated (*n* = 6 for each group). *** indicates *p* < 0.001 *versus* control.

Fifteen microRNA expression constructs from a mouse microRNA library (Figure 1A) were transfected into 4TO7 mouse breast cancer cells. The stable expression cell lines were injected into the lateral tail vein of Balb/c female mice, and the numbers of metastasis nodules in lungs were calculated 14 days after transplantation. Two of the fifteen microRNAs, mmu-miR-449a and mmu-miR-1894, decreased the formation of lung metastasis nodules by 77.1% and 86.7%, respectively, compared with that of the vector control (*n* = 6, *p* < 0.001, Figure 1A and Figure S1A,B). By contrast, mmu-miR-1935 dramatically increased the number of lung metastasis nodules by 100% (*n* = 6, *p* < 0.001, Figure 1A).

Mmu-miR-1894 was identified recently [24], and the knowledge regarding its function is very limited. It was interesting to find that mmu-miR-1894 could inhibit the metastasis and proliferation of mouse breast cancer cells (Figure S1C). We consequently focused on the function and regulation mechanisms of miR-1894 in breast cancer cells.

MiRBase (www.mirbase.org) indicated that miR-1894 could produce two mature forms, mmu-miR-1894-3p and miR-1894-5p. Mmu-miR-1894-3p and mmu-miR-1894-5p, which are mature miRNAs derived from the 3′- and 5′-strands of the precursor mmu-miR-1894, target distinct pools of genes because of their different seed sequences. The expression of miR-1894-3p and miR-1894-5p following transfection of the miR-1894 construct was shown in Figure S2. To determine the functional mature form, constructs for the expression of mmu-miR-1894-3p and mmu-miR-1894-5p were designed and 4TO7 cell lines expressing mmu-miR-1894-3p or mmu-miR-1894-5p were established (Figure 1B). The cell growth curves and metastasis assay indicated that mmu-miR-1894-3p, rather than miR-1894-5p, inhibited cell proliferation (Figure 1C) and decreased the metastasis nodes in lung tissues by 90.8% (*n* = 6, *p* < 0.001, Figure 1D,E) compared with that of the miR-neg control. The results suggested that mmu-miR-1894-3p was the functional mature form of mmu-miR-1894 against the cell proliferation and metastasis of the breast cancer cells.

### 2.2. Mmu-miR-1894-3p Down-Regulated Trim46 by Binding to the Trim46 3′-Untranslated Region (UTR)

**Figure 2:**
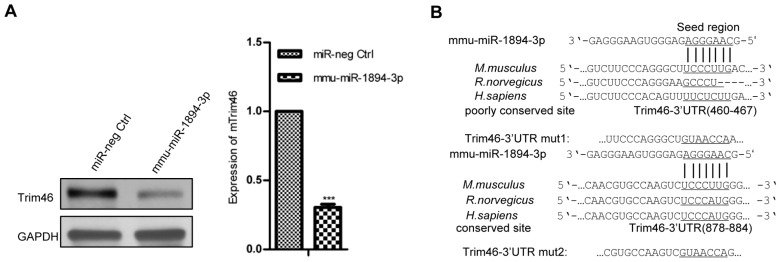

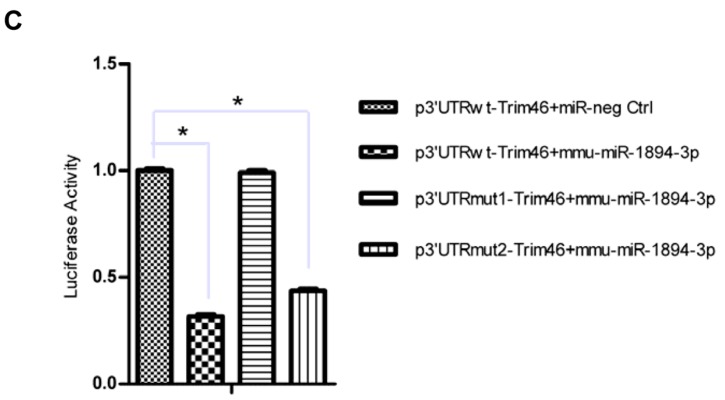
Mmu-miR-1894-3p directly targets Trim46. (**A**) The expression of Trim46 in 4TO7 cell lines was determined by western blot. GAPDH was used for normalization. *** indicates *p* < 0.001 *versus* control; (**B**) Two putative binding sites for mmu-miR-1894-3p in the 3′-UTR of Trim46 mRNA. Below are the two mutated binding sites in the 3′-UTR of Trim46 mRNA; (**C**) Dual luciferase reporter assays in 293T cells. * indicates *p* < 0.05 *versus* control.

The possible targets of mmu-miR-1894-3p were predicted using algorithms from TargetScan 6.2 (http://www.targetscan.org) and MiRanda (http://www.microrna.org). The candidate genes involved in the cell proliferation and migration were analyzed, including Eif1 [25], Hoxc8 [26], Klf4 [27], Rbm47 [28], Sema4c [29], Surf4 [30] and Trim46 [31]. The expression levels of Eif1, Hoxc8, Klf4, Rbm47, Sema4c and Surf4 did not change in the presence of mmu-miR-1894-3p (Figure S3). Only Trim46 was downregulated significantly by mmu-miR-1894-3p in 4TO7 cells (*p* < 0.001, Figure 2A).

The two possible mmu-miR-1894-3p binding sites in the sequence of the Trim46 3′-UTR were predicted using TargetScan (Figure 2B). A dual luciferase reporter assay was performed according those two possible mmu-miR-1894-3p binding sites, and the results showed that mmu-miR-1894-3p could decrease the relative luciferase activity of the p3′-UTR wt-Trim46 construct and p3′-UTR mut2-Trim46 construct (*p* < 0.05, Figure 2C), whereas the luciferase activity was not significantly attenuated in the target region of the p3′-UTR mut1-Trim46 construct (Figure 2C). These data suggest that the functional binding site of mmu-miR-1894-3p was localized on position 460–467 of Trim46 3′-UTR.

### 2.3. Trim46 Knockdown Inhibited Cell Proliferation and Metastasis in 4TO7 Mouse Breast Cancer Cells

**Figure 3:**
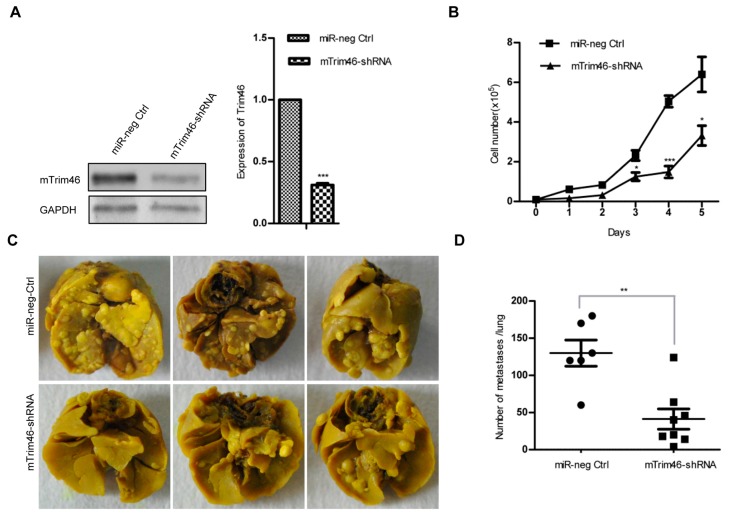
Knockdown of m-Trim46 inhibited cell proliferation and metastasis. (**A**) The expression of Trim46 in the m-Trim46-shRNA stable expression 4TO7 cell line was determined by western blot. GAPDH was used for normalization. *** indicates *p* < 0.001 *versus* control; (**B**) Growth curves of mTrim46-shRNA stable expression cell lines at indicated time. * indicates *p* < 0.05; *** indicates *p* < 0.001 *versus* control; (**C**) Representative photos of lung metastasis nodules of mTrim46-shRNA stable expression cell lines; and (**D**) The number of metastasis nodules in (**D**) was calculated (*n* = 6 for control; *n* = 8 for mTrim46-shRNA). ** indicates *p* < 0.01 *versus* control.

To determine if Trim46 was the major target of mmu-miR-1894-3p for inhibition of cancer cell metastasis, a shRNA (mTrim46-shRNA) was designed to mimic mmu-miR-1894-3p-mediated down-regulation of the expression of Trim46. A stable expression cell line of mTrim46-shRNA in 4TO7 cells was established, and the Trim46 expression was decreased by 69.1% compared with that of miR-neg control cells (*p* < 0.001, Figure 3A). The cell growth curves and metastasis assay indicated that Trim46-shRNA also inhibited cell proliferation (Figure 3B) and decreased the metastasis nodes in lung tissues (*n* = 8, *p* < 0.01, Figure 3C,D), suggesting that Trim46 was the major target of mmu-miR-1894-3p for inhibition of breast cancer cell metastasis.

### 2.4. Trim46 Was Involved in the Cell Proliferation and Migration of Human Breast Cancer Cells

**Figure 4:**
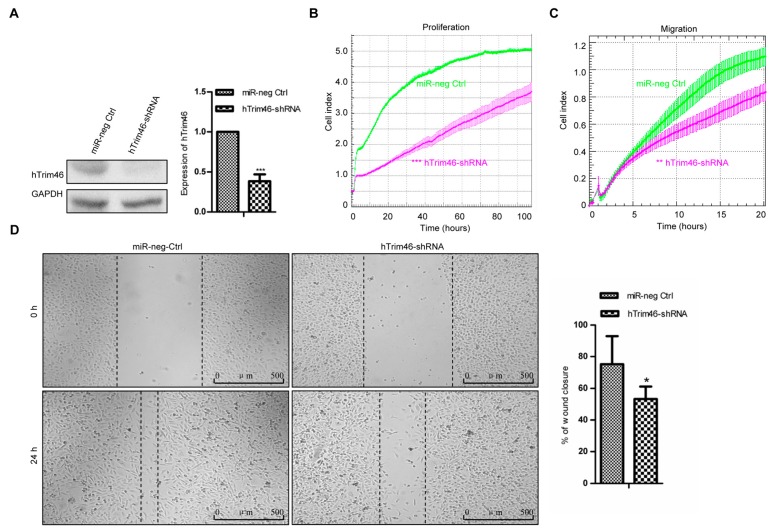
Knockdown of h-Trim46 inhibited cell proliferation and migration. (**A**) The expression of Trim46 was determined by western blot in the h-Trim46-shRNA stable expression MDA-MB-231 cells. GAPDH was used for normalization. *** indicates *p* < 0.001 *versus* control; (**B**) Cell proliferation in real time was analyzed by the xCELLigence RTCA. Shown are cell index values measured over 96 h. Error bars indicate standard deviations. *** indicates *p* < 0.001 *versus* control; (**C**) Cell migration in real time was analyzed by the xCELLigence RTCA. Shown are cell index values measured over 15 h. Error bars indicate standard deviations. ** indicates *p* < 0.01 *versus* control; and (**D**) A scratched-wound healing assay was performed in MDA-MB-231 cells with stable expression of h-Trim46-shRNA or negative control followed by photography at 0 h and at 24 h after the scratch. The statistical results are shown in the right panel. * indicates *p* < 0.05 *versus* control. Scale bar = 500 μm.

To determine if Trim46 was involved in the cell proliferation and migration of human breast cancer cells, a shRNA (hTrim46-shRNA) was designed to knock down the expression of Trim46 in human breast cancer cells. MDA-MB-231 cells lines with stable expression of hTrim46-shRNA or negative control hairpin were established, and the expression of Trim46 was decreased by 61.7% compared with that of the miR-negative control cells (*p* < 0.001, Figure 4A).

The kinetics of the proliferation and migration of the stable cell lines were evaluated by xCELLigence RTCA. Cell index profiles revealed the reduced cell proliferation (Figure 4B) and migration of the MDA-MB-231 cells expressing hTrim46-shRNA (Figure 4C). Further, the wound healing assay also revealed that hTrim46-shRNA could significantly decrease the migration rate of MDA-MB-231 breast cancer cells (*p* < 0.05, Figure 4D). These data, therefore, indicate that in addition to the regulatory function on cell proliferation, Trim46 has an impact on the migration of human breast cancer cells.

### 2.5. Trim46 Knockdown by Mmu-miR-1894-3p or shRNA Reduced Its Expression on the Cell Surface

**Figure 5:**
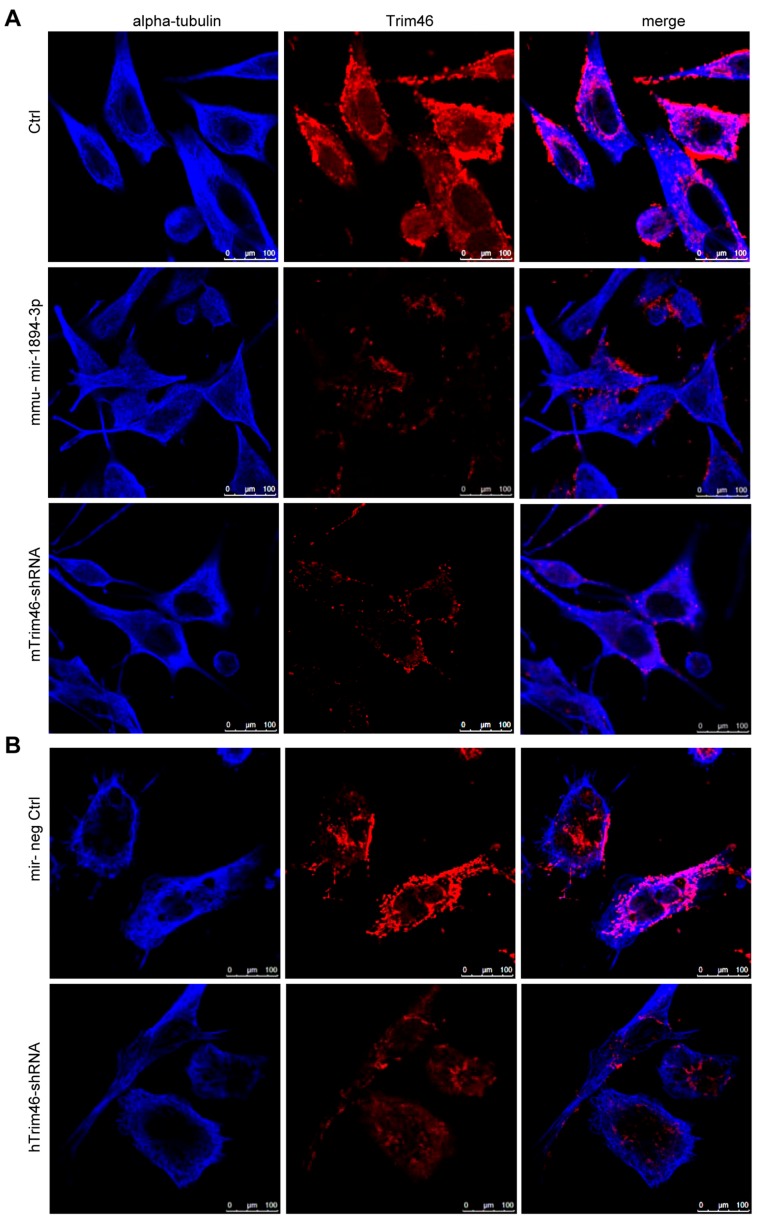
Subcellular localization of Trim46 by immunofluorescence. (**A**) 4TO7 cells expressing miR-neg control or mmu-miR-1894-3p or Trim46-shRNA were stained with α-tubulin (blue) and Trim46 (red) antibodies. Scale bars are 100 μm; and (**B**) MBA-MD-231 cells expressing miR-neg control and Trim46-shRNA cells were stained with α-tubulin (blue) and Trim46 (red) antibodies. Scale bars are 100 μm.

The localization of Trim46 on the subcellular structure was observed by the immunofluorescent staining of Trim46 and α-Tubulin. The Trim46 protein formed discrete bodies or speckles and accumulated on the apical surface of the cells (Figure 5), while it was not co-localized with the tubulin cytoskeleton. Mmu-miR-1894-3p and the shRNA knocked down the expression of Trim46 and reduced the recruitment of Trim46 to the cell membrane of both the mouse and human breast cancer cells. These suggested that the knockdown of Trim46 might disturb the interaction between the cell and the extracellular matrix (ECM).

## 3. Discussion

The final objective of our study is to identify new tumor suppressor microRNAs from 486 mouse microRNAs. This paper is part of the job and we had selected some conserved mouse miRNAs [11,12,13,14,15,16,17,18,19,20,21,22,23] with human homolog related to human cancer and several unconserved mouse miRNAs identified recently [24] to screen for new metastasis suppressor miRNA or new target genes. In the current study, we showed that three of the fifteen microRNAs, mmu-mir-449a, mmu-mir-1935 and mmu-mir-1894, had significant effects on lung metastasis of cancer cells. First, mmu-mir-449a has been shown as a tumor suppressor in endometrial cancer [16,19], gastric carcinoma [15], lung cancer [17,32], ovarian cancer [33], prostate cancer [18], *etc.* Second, mmu-mir-1935 might be more interesting, but it has been omitted from the miRbase database. Third, mmu-mir-1894 is a recently discovered mouse microRNA [24], and its biological function has not been well investigated. It is indicated that mmu-miR-1894 is close to common sites of retroviral integrations in genome instability analysis and it is predicted to likely have some role in cell homeostasis [24], which should be verified by experiments in the future.

Further, we paid attention to mmu-miR-1894, its targets and its future expectations in relationship to tumor metastasis inhibition in breast cancer cells. We found that mmu-miR-1894-3p, mature miRNA derived from the 3′-strands of the precursor mmu-miR-1894, functioned as a tumor suppressor. Mmu-miR-1894-3p directly targeted position 460–467 of the Trim46 3′-UTR and downregulated the expression of Trim46 in 4TO7 cells. The knockdown of expression of Trim46 by shRNAs also inhibited the proliferation and migration in 4TO7 cells and MDA-MB-231 human breast cancer cells. These findings suggested that the effect of knockdown of Trim46 alone was sufficient to recapitulate the effect of mmu-miR-1894 on the metastasis of the breast cancer cells in mice and that Trim46 was also involved in the proliferation and migration of human breast cancer cells.

Trim46 belongs to a large gene family with a tripartite motif (Trim) that consists of three zinc-binding domains, a RING, a B-box type 1 and a B-box type 2, and a coiled-coil region, and most of the family members are involved in cancer and development [31,34,35,36]. Trim46 (Trific) is a member of the C–I TRIM subfamily; some members of the C–I subfamily, such as MID1, MID2 and Haprin/TRIM36, were involved in the intracellular signaling at the cytoskeleton-plasma membrane interface and/or the cell adhesion [37,38,39]. However, the function of Trim46 has not been well elucidated [40]. Based on the primary sequence of Trim46, it has high similarity with Haprin/TRIM36 [37], which is involved in cell adhesion in cytomembranes and results in proper somite arrangement [41].

Our results showed that the Trim46 protein could form discrete bodies and accumulate on the apical surface of the cells. These results suggested that Trim46 was involved in the interaction between the cell and ECM, while we did not observe co-localization with microtubules. The extracellular matrix (ECM) is composed of highly variable and dynamic components that regulate cell behavior and plays a vital role as a critical contributor to tumorigenic development, growth and metastasis of breast cancer [42,43]. Of note, Trim15 was localized in the focal adhesion complex of HeLa cells, and knockdown of the expression of Trim15 could reduce the mobility of those cells [44]. Our results suggested that Trim46 might have a similar function to Trim15 in cell migration.

In addition, our study is the first study to reveal that the low expression of Trim46 significantly inhibited cell proliferation and migration of breast cancer cells. We also used TargetScan software to predict human miRNAs targeting Trim46 and found that Trim46 was the potential target of hsa-miR-96 with conserved target site at position 572–598 of Trim46 3′-UTR. Previous studies demonstrated that miR-96 functions as a tumor suppressor gene by targeting NUAK1or HERG1 in pancreatic cancer [45,46]. Thus, Trim46 might a novel target for cancer therapy, which needed to be validated in more cell lines with different cancer types in future studies.

In summary, our results show that mmu-miR-1894-3p as an important antimetastatic miRNA is associated with lung metastasis in breast cancer. Enforced expression of mmu-miR-1894-3p suppresses breast cancer cell metastasis through directly targeting Trim46. These findings suggest that Trim46 might have a therapeutic potential to suppress breast cancer metastasis.

## 4. Materials and Methods

### 4.1. Constructs

A library of 486 mouse microRNAs expression plasmids was purchased from OriGene Biotech. Of these microRNAs, fifteen constructs, including mmu-miR-487b [11,12], mmu-miR-467e [13], mmu-miR-466d [14], mmu-miR-449a [15,17,18,32,33], mmu-miR-148a [20], mmu-miR-133a-1 [21], mmu-miR-1-2-as [22], mmu-miR24-2 [23], mmu-miR-1940, mmu-miR-1935, mmu-miR-1931 [24], mmu-miR-1902, mmu-miR-1895, mmu-miR-1894 [24], and mmu-miR-1193, were examined in this study. A pCMV-MIR vector served as the control for the miRNA expression experiment.

Mmu-miR-1894-3p and mmu-miR-1894-5p expression vectors were constructed using a BLOCK-iT™ Pol II miR RNAi Expression Vector Kit with EmGFP (Invitrogen, Carlsbad, CA, USA) according to the manufacturer’s protocol. Mmu-miR-1894-3p top strand oligo 5′-TGCTGGCAAGGGAGAGGGTGAAGGGAGGTTTTGGCCACTGACTGACCTCCCTTCCCTCTCCCTTGC-3′ and bottom strand oligo 5′-CCTGGCAAGGGAGAGGGAAGGGAGGTCAGTCAGTGGCCAAAACCTCCCTTCACCCTCTCCCTTGCC-3′. Mmu-miR-1894-5p top strand oligo 5′-TGCTGCTCTCCCCTACCACCTGCCTCTGTTTTGGCCACTGACTGACAGAGGCAGGGTAGGGGAGAG-3′ and bottom strand oligo 5′-CCTGCTCTCCCCTACCCTGCCTCTGTCAGTCAGTGGCCAAAACAGAGGCAGGTGGTAGGGGAGAGC-3′. The oligonucleotides were annealed and inserted into BLOCK-iT™ Pol II miR RNAi Expression Vector. Mouse Trim46 shRNA (5′-AGTGTCGAGCTACCTTCTGTA-3′) or human Trim46 shRNA (5′-GTTTCCTTCCTGGATGCTGTT-3′) were designed using Invitrogen’s RNAi Designer (www.invitrogen.com/rnai) and were inserted into the same vector as above. The miR-negative control plasmid was also from Invitrogen.

The 3′-UTR sequences of mouse Trim46 were amplified from the normal mouse genomic DNA with the primers (restriction sites were included), Trim46 sense 5′-GTTTAAACGGGGCGGGTCTCCTTCCTG-3′ and antisense 5′-GCGGCCGCGCAGTAGCAGGCACCAGAGGC-3′. Mutations in the mmu-miR-1894-3p binding site of Trim46 were introduced by site-directed mutagenesis with the following primers: Trim46 3′-UTR mutant1 sense 5′-TGTCTTCCCAGGGCTGTAACCAACTAAGGGACTTTCC-3′ and antisense 5′-GGAAAGTCCCTTAGTTGGTTACAGCCCTGGGAAGACA-3′, Trim46 3′-UTR mutant2 sense 5′-CCAACGTGCCAAGTCGTAACCAGGAACTCAGTTAAGG-3′ and 5′-CCTTAACTGAGTTCCTGGTTACGACTTGGCACGTTGG-3′. The wild and mutant 3′-UTR sequences were introduced downstream of the *Renilla* luciferase stop codon in the *XhoI*/*NotI* cloning sites of the psiCHECK 2 vector (Promega, Madison, WI, USA), respectively.

### 4.2. Cell Culture

4TO7 and MDA-MB-231 cells were obtained from ATCC (Manassas, VA, USA) and were freshly recovered from liquid nitrogen. The breast cancer cells were maintained in Dulbecco’s modified Eagle’s medium (DMEM) (Gibco, Grand Island, NY, USA) supplemented with 10% fetal bovine serum (FBS, Gibco), 1% l-glutamine, 100 U/mL penicillin, and 100 mg/mL streptomycin. All cells were grown and maintained at 37 °C in a 5% CO_2_ humidified incubator (Sanyo, Osaka, Japan).

Plasmids transfection was performed using Lipofectamine 2000 (Invitrogen) following the manufacturer’s instructions. For the stable transfection of microRNAs, the cells were selected with 500 μg/mL neomycin (AMRESCO, Solon, OH, USA) and sorted by a FACSAria I cell sorter (Becton-Dickinson, Franklin Lakes, NJ, USA). For the stable transfection of microRNA or shRNA constructs, the cells were selected with 3 μg/mL blasticidin (Sigma-Aldrich, St. Louis, MO, USA) and sorted by flow cytometry.

### 4.3. qRT-PCR

Total RNA was extracted from cells using mirVana™ miRNA Isolation Kit (Ambion, Austin, TX, USA) and treated with DNase I (Ambion) to obtain DNA-free RNA, according to the manufacturer’s protocol. Reverse transcription of RNA into cDNA was performed using a miScript II RT Kit (Qiagen, Valencia, CA, USA). The RT products were subsequently amplified with mature miRNA-specific miScript Primer Assays (Qiagen) using the Applied Biosystems StepOne™ Real-Time PCR system (Applied Biosystems, Foster City, CA, USA). The PCR was performed at 95 °C for 15 min, followed by 40 cycles of 95 °C for 15 s, 55 °C for 30 s and 70 °C for 30 s. U6 small nuclear RNA (Hs_RNU6-2_11 miScript Primer Assay) was used as an internal control for normalization. The relative expression was calculated using the comparative ΔΔ*C*_t_ method and fold change calculated using 2^−ΔΔ*C*t^.

### 4.4. Western Blot

The cells were lysed in Radio-Immunoprecipitation Assay (RIPA) lysis buffer containing phenylmethanesulfonyl fluoride (PMSF) and separated on sodium dodecyl sulfate polyacrylamide gel electropheresis (SDS-PAGE). The proteins were transferred to a nitrocellulose (NC) membrane (Millipore, Waltham, MA, USA) and probed with anti-Trim46 antibody (1:1000, Proteintech, Chicago, IL, USA). The primary antibody was detected with anti-rabbit HRP-conjugated secondary antibodies (Santa Cruz, Dallas, TX, USA). Horseradish peroxidase (HRP)-conjugated GAPDH antibody was used for normalization. The NC membrane was visualized using ECL (Santa Cruz) and exposed to X-ray film (Kodak, Rochester, NY, USA).

### 4.5. Target Prediction

The analysis of mmu-miR-1894-3p predicted targets was determined using the algorithms of TargetScan mouse release 6.0 (http://www.targetscan.org/)and MiRanda (http://www.microrna.org). According to these algorithms [47], the intersection of both resulted in a list of 31 common genes as potential targets of mmu-miR-1894-3p, depicted in Table S1 in the supplemental material.

### 4.6. Dual-Luciferase Reporter Assays

The cells were seeded in triplicate in 24-well plates one day before transfection for the luciferase assays. Wt (p3′-UTR wt-Trim46) or mut 3′-UTR (p3′-UTR mut1-Trim46 and p3′-UTR mut2-Trim46) constructs were co-transfected with the mmu-miR-1894-3p expression vector or negative control into HEK-293T cells using Lipofectamine 2000 reagent (Invitrogen). After 48 h of transfection, the cells were harvested and lysed and the luciferase activity was assayed using the Dual Luciferase Reporter Assay System (Promega) according to the manufacturer’s instructions. The *firefly* luciferase values were normalized to *Renilla*, and the relative ratios of *firefly* to *Renilla* activity were reported. Three independent experiments were performed, and the data are presented as the mean ± Standard Error of Mean (SEM).

### 4.7. In vivo Metastasis Assays

For *in vivo* metastasis assays, breast cancer cells were re-suspended in 0.1 mL of phosphate buffer saline (PBS) and injected into the lateral tail vein of 6-week-old Balb/c female mice (6 per group, 1 × 10^6^ cells per mouse were used for the first animal experiment, 5 × 10^5^ cells per mouse for the followed study). Mice were sacrificed after two weeks. Lungs were harvested and stained with Bouin’s solution. Metastatic nodes were observed by naked eye. All of the research involving animals complied with protocols approved by the Institutional Animal Care and Use Committee of the Chinese Institute of Laboratory Animal Science (ILAS-GC-2015-002).

### 4.8. RTCA Assays

Real-time cell analyzer (RTCA, xCELLigence Roche, Basel, Switzerland) was used to assess cellular proliferation and migration in real time on a cell culture level. For proliferation assay, cells (1 × 10^4^ per well) were introduced to E-Plates (Roche), and the cell growth curves was recorded on the xCELLigence System every 15 m for 96 h. For migration assay, cells (4 × 10^4^ per well) were starved in serum-free medium for 24 h and seeded in RTCA CIM-16 plates (Roche) in serum-free medium. Full growth medium was used as a chemoattractant in the lower chamber. The cell index values were monitored every 10 m for 24 h.

### 4.9. Immunofluorescence

Transfected cells were grown on coverslips for 18 h in DMEM plus 10% fetal calf serum and fixed using 4% paraformaldehyde. Microtubule staining was performed post-fixation using an anti-α-tubulin primary antibody (1:200, CST, Danvers, MA, USA) and a DyLight 405-labeled anti-mouse secondary antibody (KPL, Gaithersburg, MD, USA). Trim46 was detected using a rabbit anti-Trim46 antibody (1:25, Proteintech, Chicago, IL, USA) and a Donkey anti-Rabbit IgG (H+L) Secondary Antibody, Alexa Fluor^®^ 555 conjugate (Thermo Fisher Scientific, Rockford, IL, USA). Images were captured using a Leica TCS SPE laser scanning confocal microscope (Leica Microsystems GmbH, Mannheim, Germany).

### 4.10. Wound Healing Assay

Cells (5 × 10^5^ cells/well) were seeded in a 6-well plate and allowed to form a monolayer overnight. A 200-μL sterile plastic tip was used to create a wound line across the surface of the plates. Then, the suspension cells were removed by washing with PBS three times. Cells were incubated in serum-free medium. Photos were taken immediately (*t* = 0 h) or 24 h later (*t* = 24 h) under a microscope (Leica).

### 4.11. Statistical Analysis

All data are shown as mean ± SEM unless otherwise noted; Student’s *t*-test was used for statistical analysis when only two groups were tested, and one-way analysis of variance was used to compare multiple groups. In all cases, *p* < 0.05 was considered to be statistically significant.

## Supplementary Materials

Supplementary materials can be found at http://www.mdpi.com/1422-0067/17/4/609/s1.
