# Supplementary material for: Mmu-miR-1894-3p Inhibits Cell Proliferation and Migration of Breast Cancer Cells by Targeting Trim46
Source: Int J Mol Sci. 2016 Apr 22;17(4):609. doi: 10.3390/ijms17040609 (PMC4849059; doi:10.3390/ijms17040609)
Supplement: Supplementary file 1 [file ijms-17-00609-s001.pdf]

Supplementary Materials: Mmu-miR-1894-3p Inhibits Cell Proliferation and Migration of Breast Cancer Cells by Targeting Trim46

Li Zhang, Xiaoying Li, Wei Dong, Caixian Sun, Deyu Guo and Lianfeng Zhang

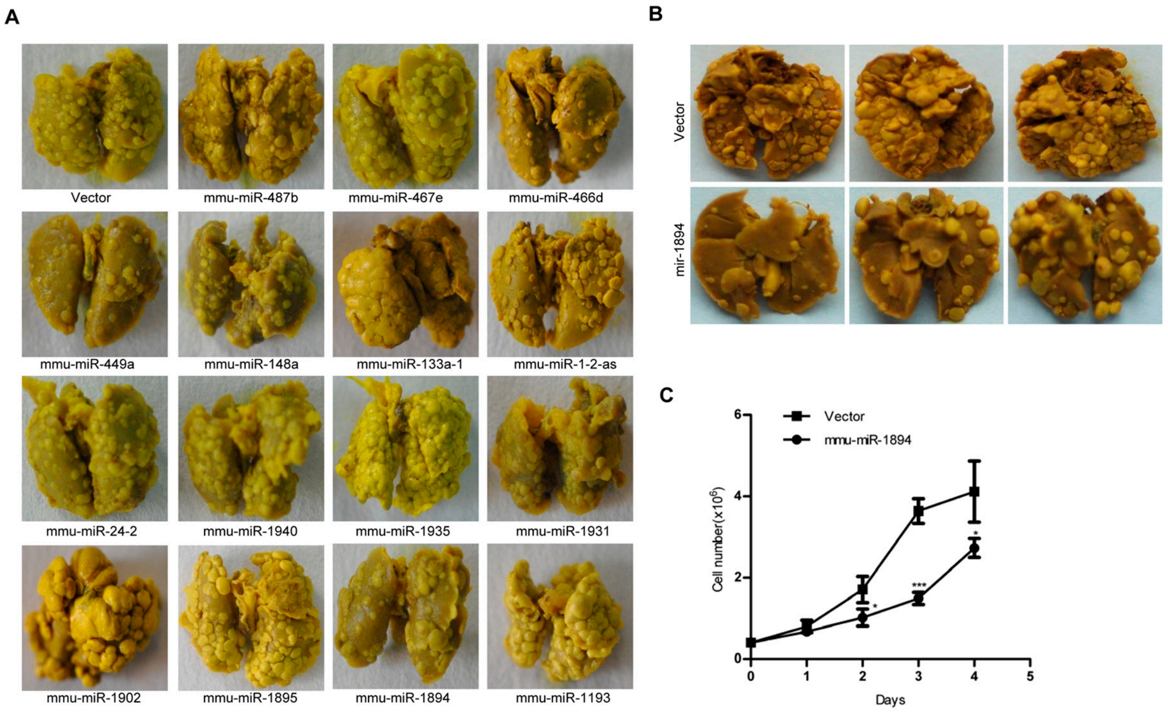

Figure S1. Fifteen mouse microRNAs and lung metastasis assay. (**A**) 4TO7 mouse breast cancer cells were transfected with empty vector or fifteen individual microRNA expression plasmids, including miR-487b, miR-467e, miR-466d, miR-449a, miR-148a, miR-133a-1, miR-1-2-as, miR-24-2, miR-1940, miR-1935, miR-1931, miR-1902, miR-1895, miR-1894, and miR-1193. Stable cell lines were generated and 1×10^6 cells were injected into the tail veins of Balb/c female mice (n = 6 for each group). Two weeks later, the mice were sacrificed; the lungs were fixed in Bouin's solution and photographed. Shown are the representative photos for lung metastasis nodules (one lung for each microRNA); (**B**) Representative photos for lung metastasis of 4TO7 cells expressing miR-1894 and the vector control; and (**C**) 4TO7 cells expressing miR-1894 and vector control were seeded into 24-well plates at 1×10^4 cells/well and counted using Trypan Blue after 24, 48, 72 and 96 h. Growth curves were made from data of cell counting for four days. * indicates $p < 0.05$; *** indicates $p < 0.001$ *versus* vector control.

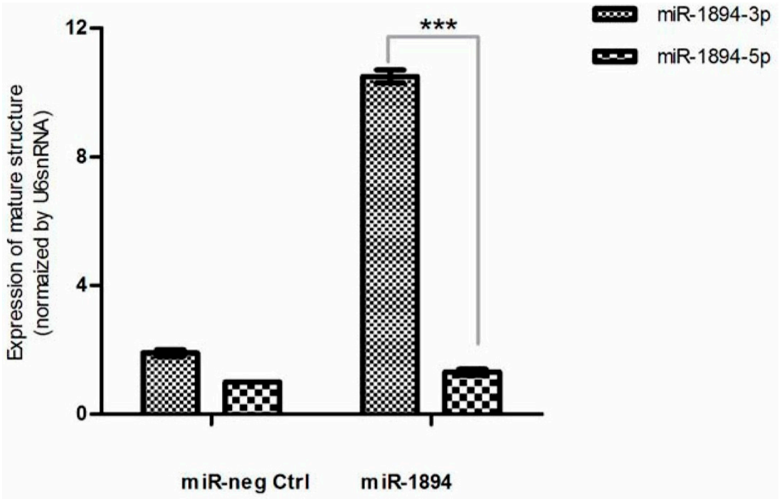

Figure S2. The expression level of two mature structures of miR-1894 in 4TO7 mouse breast cancer cells. miR-1894 produces two mature structure forms, miR-1894-3p and miR-1894-5p. The expression of miR-1894-3p and miR-1894-5p in 4TO7 cells expressing miR-1894 and the vector control was detected by real-time PCR. *** indicates $p < 0.001$ miR-1894-3p *versus* miR-1894-5p.

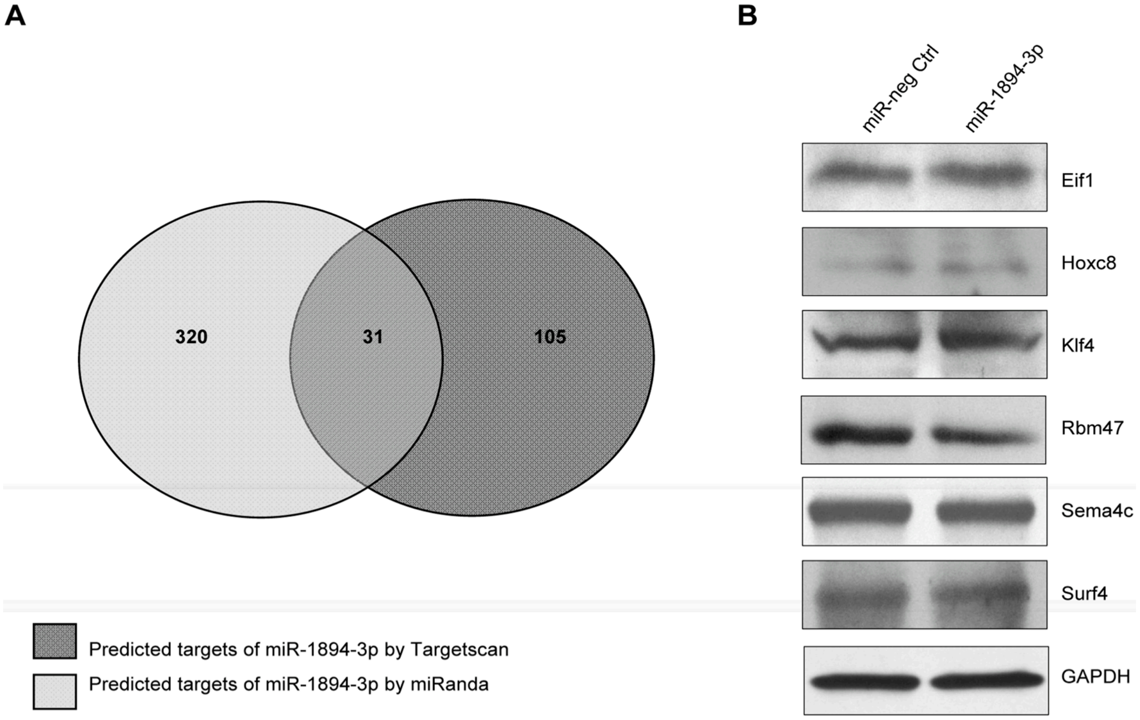

Figure S3. Identification of pontential target genes. (**A**) miR-1894-3p targets were predicted using the TargetScan and miRanda programs, and the lists of predicted targets were merged, resulting in 31 genes as candidates for direct targeting by miR-1894-3p.B. Seven genes (Eif1, Hoxc8, Klf4, Rbm47, Sema4c, Surf4 and Trim46) were selected as candidate targets of miR-1894-3p; and (**B**) Immunoblotting was carried out with antibodies specific for Eif1(1:200, Santa Cruz), HOXC8 (mAb; 1:1000; Abcam), KLF4 (1:1000, Abcam), Rbm47 (1:1000, Novus Biologicals), Sema4c (1:500, Bioss) SURF4-C-terminal (1:1000, Abcam), Trim46 (1:2000, Proteintech), Primary antibodies were visualized with anti-rabbit HRP conjugated secondary antibodies. GAPDH was used for normalization.

Table S1. Thirty-one potential targets predicted by TargetScan and MiRanda programs.

No.	Representative Transcript	Gene Name
1	NM_175123	*RIKEN cDNA 1110051M20* gene
2	NM_001081400	*RIKEN cDNA 1810013L24* gene
3	NM_002892	*AT rich interactive domain 4A (RBP1-like)*
4	NM_001126054	*Calcium/calmodulin-dependent serine protein kinase (MAGUK family)*
5	NM_001173982	*Carbohydrate (chondroitin 4) sulfotransferase 11*
6	NM_008176	*Chemoki ne (C-X- C motif) ligand 1*
7	NM_005801	*Eukaryotic translation initiation factor 1*
8	NM_001013415	*F-box and WD repeat domain containing 7*
9	NM_022658	*Homeobox C8*
10	NM_001034914	*Kcnip3 Kv channel interacting protein 3, calsenilin*
11	NM_000899	*KIT ligand*
12	NM_004235	*Kruppel-like factor 4 (gut)*
13	NM_001080774	*Myosin IC*
14	NM_002522	*Nptx1 neuronal pentraxin I*
15	NM_004161	*RAB1A, member RAS oncogene family*
16	NM_001098634	*RNA binding motif protein 47*
17	NM_172612	*Rnd1Rho family GTPase 1*
18	NM_017789	*Sema domain, immunoglobulin domain (Ig), transmembrane domain (TM) and short cytoplasmic domain, (semaphorin) 4C*
19	NM_001172700	*Shroom family member 1*
20	NM_001128225	*Solute carrier family 39 (zinc transporter), member 13*
21	NM_000342	*Solute carrier family 4, anion exchanger, member 1 (erythrocyte membrane protein band 3, Diego blood group)*
22	NM_011512	*Surfeit* gene 4
23	NM_006950	*Synapsin I*
24	NM_015527	*TBC1 domain family, member 10B*
25	NM_001033422	*THO complex 2*
26	NM_001126339	*Thiamine triphosphatase*
27	NM_001039466	*Tripartite motif-containing 46*
28	NM_025692	*Ubiquitin-like modifier activating enzyme5*
29	NM_003680	*Tyrosyl-tRNA synthetase*
30	NM_152758	*YTH domain family, member 3*
31	NM_014827	*Zinc finger CCCH-type containing 11A*
